# Zika virus as an oncolytic treatment of human neuroblastoma cells requires CD24

**DOI:** 10.1371/journal.pone.0200358

**Published:** 2018-07-25

**Authors:** Joseph Mazar, Yujia Li, Amy Rosado, Peter Phelan, Kritika Kedarinath, Griffith D. Parks, Kenneth A. Alexander, Tamarah J. Westmoreland

**Affiliations:** 1 Department of Biomedical Research, Nemours Children’s Hospital, Orlando, Florida, United States of America; 2 Burnett School of Biological Sciences, University of Central Florida College of Medicine, Orlando, Florida, United States of America; CEA, FRANCE

## Abstract

Neuroblastoma is the second most common childhood tumor. Survival is poor even with intensive therapy. In a search for therapies to neuroblastoma, we assessed the oncolytic potential of Zika virus. Zika virus is an emerging mosquito-borne pathogen unique among flaviviruses because of its association with congenital defects. Recent studies have shown that neuronal progenitor cells are likely the human target of Zika virus. Neuroblastoma has been shown to be responsive to infection. In this study, we show that neuroblastoma cells are widely permissive to Zika infection, revealing extensive cytopathic effects (CPE) and producing high titers of virus. However, a single cell line appeared poorly responsive to infection, producing undetectable levels of non-structural protein 1 (NS1), limited CPE, and low virus titers. A comparison of these poorly permissive cells to highly permissive neuroblastoma cells revealed a dramatic loss in the expression of the cell surface glycoprotein CD24 in poorly permissive cells. Complementation of CD24 expression in these cells led to the production of detectable levels of NS1 expression after infection with Zika, as well as dramatic increases in viral titers and CPE. Complementary studies using the Zika virus index strain and a north African isolate confirmed these phenotypes. These results suggest a possible role for CD24 in host cell specificity by Zika virus and offer a potential therapeutic target for its treatment. In addition, Zika viral therapy can serve as an adjunctive treatment for neuroblastoma by targeting tumor cells that can lead to recurrent disease and treatment failure.

## Introduction

Neuroblastoma is a childhood cancer that affects approximately 1/7000 children [1], commonly developing along the sympathetic nervous system or adrenal glands. The incidence of neuroblastoma is about 10.54 cases per 1 million per year in children younger than 15 years [2]. In the Unites States, neuroblastomas account for 6% of all childhood cancers but cause a disproportionally high 15% of all childhood cancer deaths [3, 4]. While a small subset of neuroblastoma will undergo spontaneous regression, most neuroblastoma progress relentlessly despite aggressive chemotherapy, radiation, and even autologous transplantation. The 5-year survival rates for neuroblastoma in children ages 1–14 years is 68% [5]. Unfortunately, long-term survival of patients with high-risk tumor phenotypes is less than 40% [6, 7]. Given such high mortality, there is a substantial need to identify new therapies for high-risk neuroblastoma.

The use of viruses for the therapeutic treatment of cancer was first suggested at the beginning of the twentieth century. Hepatitis viruses were among the first oncolytic viruses to be used in the treatment of therapy of Hodgkin's disease in the later 1940s, leading to brief remission of the disease following viral inoculation [8]. Similar results were observed in a patient suffering from monocytic leukemia naturally infected with Epstein-Barr virus [9]. Later clinical trials in the 1950s investigated West Nile virus, adenovirus, mumps virus, and various enteroviruses for their oncolytic properties [10–12]. However, nearly every virus studied exhibited a degree of neurotropism matching or exceeding the viral tumor tropism. Recently developed oncolytic paramyxoviruses, herpes viruses, picornaviruses, and poxviruses, are more tumor specific [13–15]. However, the pathogenicity of these viruses often precluded therapeutic use. Although the majority of the recent oncolytic viruses in clinical testing are typically genetically engineered derivatives of previously studied pathogens, no prime therapeutic candidate has arisen [16–18]. As such, there continues to be a need for oncolytic viruses that exhibit high tumor specificity.

Zika viruses are neurotropic members of the virus family *Flaviviridae* [19]. In children and non-pregnant adults, eighty percent of Zika virus infections are asymptomatic and the vast majority of symptomatic Zika virus infections are marked only by self-limiting rash, conjunctivitis, and fever [20]. In contrast to the mild clinical courses of Zika virus infection in children and adults, acute maternal-fetal Zika virus infection is now well recognized as a cause of severe fetal microcephaly, arthrogryposis, and retinitis, as well as spontaneous abortion [21–25]. Recognizing that microcephaly cases in Brazil were associated with the ongoing Zika virus epidemic, several investigators have now demonstrated that infection of neuronal progenitor cells underlies the congenital Zika virus syndrome [26–28].

Recently, Hughes *et al*. and Luplertlop *et al*. demonstrated that Zika viruses cause lytic infection in several (but not all) neuroblastoma cell lines and Zhu *et al* showed recently that mice with glioblastomas xenografts survived significantly longer when the tumor was inoculated with Zika virus [29–31]. Given the benign natural history of Zika virus infection in non-pregnant individuals, these observations that Zika viruses cause lytic infection of cultured neuroblastoma cells and that xenografted glioblastoma cells can be treated *in vivo* with Zika viruses raises the prospect that Zika viruses might be used as directed therapy for the treatment of neuroblastomas and glioblastomas. A therapeutically-administered Zika virus could provide a targeted treatment for residual disease in children with neuroblastoma. Furthermore, the minimal pathogenic effects of natural Zika virus infection in children offers the prospect of a therapy devoid of the long-term toxic effects of standard surgery, chemotherapy, and radiation. In order to determine the potential of Zika virus as an oncolytic therapy, we investigated its ability to infect an array of human neuroblastoma cell lines and measured the pathogenic effects of infection.

Our findings indicate that neuroblastoma cells are permissive to Zika virus infection, and that cytopathic effects are induced by this infection. Furthermore, our data raise the prospect that Zika viruses may be useful for the oncolytic therapy of other pediatric tumors.

## Materials and methods

### Cell lines, viruses, and culture conditions

IMR-32 cells were cultured in Minimum Essential Medium (MEM) Alpha + GlutaMAX™ [Gibco Life Sciences] supplemented with 10% fetal bovine serum [FBS]. SK-N-AS cells were cultured in Dulbecco’s modified Eagle’s medium (DMEM) [Gibco Life Sciences] supplemented with 10% FBS and 1% non-essential amino acids (NEAA). Both IMR-32 and SK-N-AS cells were purchased from ATCC. CHLA-42 cells were cultured in HyClone Iscove’s Modified Dulbecco’s Medium (IMDM) [GE Healthcare Life Sciences] supplemented with 20% FBS and 1X ITS (5 μg/mL insulin, 5 μg/mL transferrin, 5 ng/mL selenous acid). LA-N-6, SK-N-Be(1), and SMS-KAN cells were cultured in HyClone RPMI-1640 [GE Healthcare Life Sciences] supplemented with 10% FBS. CHLA-42, LA-N-6, SK-N-Be(1), and SMS-KAN cells were all obtained from the Children’s Oncology Group (Columbus, OH). CHME3 cells were acquired and utilized with the permission of the laboratory of Dr. Ali Amara (Institut Universitaire d'Hématologie, Paris, France) and cultured in Dulbecco’s modified Eagle’s medium (DMEM) [Gibco Life Sciences] supplemented with 10% FBS. All cells were incubated and maintained at 37°C in 5% CO_2_.

All Zika viruses were obtained from American Type Culture Collection (ATCC) and were propagated in Vero cells following low multiplicity of infection (0.01 MOI). Strain PRVABC59 (ATCC^®^ VR-1843) is a Puerto Rican strain isolated from a human in 2015. The prototype strain MR766 (ATCC^®^ VR-84) was isolated originally from the blood of a sentinel monkey in Uganda in 1947. The IBH30656 strain (ATCC^®^ VR-1839) was isolated from the blood of a human in Ibadan Nigeria in 1968. Virus stocks were titered by an agar overlay plaque assay on Vero cell monolayers using established procedures (Parks et al., 2001 PMID:11160725). Alternatively, virus samples in culture media were serially diluted and used to infect Vero cells in a 96 well dish. After 4 days incubation, monolayers were scored for cytopathic effect by staining with crystal violet. Titers are expressed as a median tissue culture infectivity dose (TCID50) using the method of Reed and Muench [32].

### Cell line infection and cell viability assays

8 x 10^3^ cells of each cell line were seeded into 12 wells of flat bottom 96-well tissue culture treated plates and allowed to attach overnight. The following day, each cell line was either infected with a multiplicity of infection (MOI) = 10 of Zika virus strain PRVABC59, or treated with non-infected conditioned media (controls). Six plates were prepared simultaneously for each cell line (allowing for assays on Days 0, 2, 4, 6, 8, & 10). All cells were maintained at 37°C in 5% CO_2._ Two hours after infection, the first plate was assessed using the CellTiter 96® AQueous One Solution Cell Proliferation (MTS) assay (Promega Corp.) according to the manufacturer’s instructions. Infected wells were supplemented with 50 uL of fresh media on days 4 and 8 post-infection. Uninfected cells were split 50% on days 4, 6, and 8 to avoid overcrowding and cell death. All samples were read on a SpectraMax M5 (Molecular Devices Corp.) system at a wavelength of 490 nm using SoftMax Pro (version 6.2.1) software. Plates were examined again at each time point (days 2, 4, 6, 8, & 10 post-infection).

### Western blot analyses of Zika-infected cell lines

2.5 x 10^5^ cells were placed into 12-well tissue culture plates and allowed to attach overnight. The following day, each cell line was either infected with an MOI = 10 of Zika virus strain PRVABC59 or treated with non-infected conditioned media (controls). The cells were incubated for 4 days at 37°C in 5% CO_2._ After 4 days, the cells were collected and counted. 2 x 10^5^ cells of each cell sample were boiled in sample buffer (SDS/β-ME) and proteins separated by electrophoresis on 10% Tris-Glycine denaturing polyacrylamide gels. Proteins were transferred to nitrocellulose membranes (0.2 um, BioRad, Cat# 1620112) and probed with the following primary antibodies: Anti-Zika virus NS1 (non-structural protein 1) (One World Lab, Cat# 55964) at 1/200, anti-Zika virus Envelope (Env) (GeneTex, Cat# GTX133314) at 1/1000, and anti-GAPDH (Santa Cruz, FL-335) at 1/2000. Blots were probed with horseradish peroxidase-conjugated secondary antibodies (Invitrogen, Goat anti-Mouse, Cat# 62–6520, Goat anti-Rb, Cat# 65–6120) and visualized with ECL chemiluminescence (Pierce).

### Viral Titer (TCID50) assays

Neuroblastoma cells (IMR-32, SK-N-AS, SK-N-AS/VO, SK-N-AS/CD24 variant 1, or SK-N-AS/CD24 variant 7) were seeded in triplicate into wells of a 24-well plate at a density of 6 x 10^4^ cells per well, treated with an MOI = 10 of Zika virus strain PRVABC59, and incubated overnight. Following the overnight infection (16-18h; referred to as Day 1 post-initial infection), the media was removed to clear away any residual virus from the initial treatment, the cells were washed with PBS, and fresh media was added. At Day 2 and 3 following infection, 100 uL of media was harvested and collected from each sample. The remaining media in the wells was removed, the cells were washed with PBS, and collected using Accumax (Thermo Fisher Scientific). These collected cells were used for immunocytochemistry labeling (see below).

To measure viral titers within the culture media, 1 x 10^4^ Vero cells were seeded as a monolayer into 96-well plates. The plated Vero cells were infected with prepared serial 10-fold dilutions of virus collected from Day 2 and Day 3 of the previously infected neuroblastoma cells. Serial dilutions ranging between 10^−2^ to 10^−8^ were made. This was performed in replicates of n = 6 wells (per dilution). Four days post-infection, the wells were scored for cytopathic effects by bright field microscopy. Vero cells were then fixed with 7.4% formaldehyde and stained with crystal violet to assess monolayer integrity. TCID50 titers were determined using the method of Reed and Muench [32].

### Immunocytochemistry of Zika virus infected neuroblastoma cells

A total of 5 x 10^4^ cells of each neuroblastoma cell line and derivative (IMR-32, SK-N-AS, SK-N-AS/VO, SK-N-AS /CD24 v1, and SK-N-AS /CD24 v7) were spotted on glass slides by cytospin. The slides were washed with PBS and stained using the Thermo Shandon Sequenza system, with reagents applied manually onto slides using both gravity and capillary effects. The slides were incubated with GeneTex Anti-Zika Envelope antibody (GTX133314) at a dilution of 1:100 for 1 h. A 2^o^ anti-Rb Alexa Fluor 594-labeled antibody was used at a dilution of 1:250, and the mixtures were incubated on the slides for 30 min. The slides were incubated for 5 min with Molecular Probes DAPI (D1306) prior to cover-slipping using Molecular Probes Prolong Gold. All incubations were performed at room temperature. All slides were scanned using a Nikon A1R VAAS laser point- and resonant-scanning confocal microscope (using a single photon Ar-ion laser at a magnification of 60x with a 4x zoom). Z-stacking was performed using 32 overlapping scans compiled using NIS-Elements 4.5 imaging software.

### Quantitative real-time PCR

Total RNA was isolated from cells using an RNeasy Mini Kit (Qiagen) and RNA concentrations were determined by UV spectrophotometry. Reverse transcription (RT) reactions were used to convert ~ 1.0 μg of total RNA into cDNA using the Applied Biosystems High Capacity cDNA RT kit (Thermo Fisher Scientific). Reaction volumes were then brought to 100 μl with nuclease-free water. Quantitative real-time PCR (qPCR) was performed by using the CFX384 Touch Real-Time PCR Detection System (Bio-Rad Laboratories, Hercules, CA). Gene-specific primers for quantitative real-time PCR were designed from their respective gene sequences using PrimerQuest (Integrated DNA Technologies) to generate sequences for PCR amplicons of 75 to 150 nucleotides that span exon-exon junctions. Gene-specific qPCR primer sequences used were as follows: GAPDH, sense primer, 5’-ACATCGCTCAGACACCATG-3’, and anti-sense primer, 5’-TGTAGTTGAGGTCAATGAAGGG-3’; AXL sense primer, 5’-GTCCTCATCTTGGCTCTCTTC-3’, and anti-sense primer, 5’-GACTACCAGTTCACCTCTTTCC-3’; CD24 variant 001, sense primer, 5’-CTGCTGCTGCTGGCACTGCTCC-3’, and anti-sense primer, 5’-GGGGCCAACCCAGAGTTGGAAG-3’; and CD24 variant 007, sense primer, 5’-CTGGGCCTGGGAGACCCTAGCG-3’, and anti-sense primer, 5’-GGGGCCAACCCAGAGTTGGAAG-3’. Synthetic double-stranded, linear DNA gBlock gene fragments (Integrated DNA Technologies) corresponding to each gene-specific PCR amplicon were designed for qPCR standards. Standard curve copy numbers were calculated using the precise molecular weight of each dsDNA gBlock. The 384-well real-time PCR format included duplicate 10-fold dilutions of the linear dsDNA gene Block standards ranging from 2 x 10^7^ to 2 x 10^1^ copies per qPCR reaction. Human GAPDH was used to normalize the starting quantity of RNA. Reactions were performed in a 10-μl volume comprised of 2 μl of cDNA reaction, 5.0 μl of 2x SsoFast™ EvaGreen® Supermix (Bio-Rad), and 500 nM concentrations of each primer. The 2-step cycling parameters were 95°C for 30 sec to activate the polymerase, followed by 40 cycles of 95°C for 5 sec and 60°C for 5 sec. Fluorescence measurements were taken at each cycle during the 60°C annealing/extension step. Melt curve analysis of generated PCR amplicons was performed upon completion of the 40 amplification cycles, which consisted of a 65°C to 95°C gradient at 0.5°C increments for 2 sec plus fluorescence measurements. The copy number for each reaction was calculated by the CFX Manager 3.1 software (Bio-Rad). Copy number values were normalized to the corresponding GAPDH values to determine the relative copy number.

### Construction of CD24 recombinant expression vectors

Total RNA was isolated from IMR-32 cells using an RNeasy Mini Kit (Qiagen). Purified RNA was reverse-transcribed using M-MLV reverse transcriptase (ThermoFisher Scientific, Cat# 4368814). The resulting rcDNA was then used as a template for PCR amplification using GoTaq (Promega). The PCR primers were designed as follows: CD24–001 ORF For (BamHI)–tggatccatgggcagagcaatggtggcc, or CD24–007 ORF For (BamHI)–tggatccatggtgggacgattctgtccc and CD24 ORF Rev (EcoRI)–agaattcttaagagtagagatgcagaagagagagtg. Both PCR products were gel purified (QIAquick Gel Extraction kit, Qiagen), TOPO-cloned into pCR4-TOPO (Life Technologies), transformed into Top10 Chem comp cells and then plated onto LB Amp plates (100 ug/mL). Colonies were grown in LB Amp (100 ug/mL) overnight at 37°C. Plasmids were harvested by miniprep (QIAprep Spin Miniprep kit, Qiagen). All clones were sequenced (Retrogen), and then analyzed using VectorNTi and AlignX (Life Technologies). Both CD24 splice variants 001 and 007 were sub-cloned into pcDNA6/V5-HisA by restriction digestion using BamHI and EcoRI and ligated using T4 Ligase (NEB, Inc.). Insertion of the 001 and 007 splice variants into the final clones was confirmed by restriction digestion.

### Western blot analysis of native CD24 expression in neuroblastoma cell lines

2 x 10^5^ cells of six neuroblastoma cell lines [IMR-32, SMS-KAN, SK-N-AS, LA-N-6, SK-N-Be(1), and CHLA-42] and Vero cells were acquired and counted. Cells were then boiled under denaturing conditions and proteins separated by electrophoresis on 12% Tris-Glycine denaturing polyacrylamide gels. Proteins were transferred, probed, and visualized as above. The following primary antibodies were used: anti-CD24 (Monoclonal SN3, Cat# MA5-11828, ThermoFisher) at 1/200 and anti-GAPDH (Santa Cruz, FL-335) at 1/2000.

### Zika virus infection of IMR-32 cells after transient transfection of anti-CD24 siRNA

IMR-32 cells were seeded into single wells of a 6-well plate at a density of 2.5 x 10^5^ cells per well and transfected with 50 uM of CD24 Silencer Select Pre-designed siRNA (Cat # 4392420, ID: s2616) or Silencer Select Negative Control siRNA #1 (Cat # 4390843). The transfection was allowed to continue 6 hours, after which the media was were removed, the cells washed with PBS, and fresh media added. 48 hours after transfection, the cells were lifted (Accumax, Thermo Fisher Scientific), counted, and 2.8 x 10^5^ cells of each sample were plated in two wells of a 12-well tissue culture plate. Each sample was then either infected with MOI = 10 of Zika virus strain PRVABC59 or treated with non-infected conditioned media (control). The samples were then incubated for 4 days at 37°C in 5% CO_2_ after which they were acquired and counted. Anti-Envelope and anti-NS-1 Western blot analysis was performed once again (as above).

### Construction of SK-N-AS cells stably-expressing CD24 variants 1 and 7

SK-N-AS cells were seeded into single wells of a 6-well plate at a density of 2.5 x 10^5^ cells per well and transfected using Fugene 6 (Promega Corp.) with 2 μg of either pcDNA6/V5-HisA (Vector Only), pcDNA6/CD24-v001 or pcDNA6/CD24-v007. The transfection was allowed to continue 6 hours, after which the media was removed, the cells were washed with PBS, and fresh media was added. 24 hours after transfection, the cells began selection at 6 ug/mL with Blasticidin (Life Technologies Corp.). Selection continued for 10 days until individual colonies could be isolated.

### Confirmation of stable expression of CD24 variants 1 and 7 in SK-N-AS cells

2.5 x 10^5^ cells of each selected SK-N-AS sample (to include WT SK-N-AS, SK-N-AS/VO, SK-N-AS/CD24-v001, and SK-N-AS/CD24-v007) were acquired and counted. These cells were then boiled in sample buffer (SDS/β-ME) and proteins separated by electrophoresis on an 18% Tris-Glycine denaturing polyacrylamide gels. Proteins were transferred probed, and visualized as above. The following primary antibodies were used: anti-CD24 (Monoclonal SN3, Cat# MA5-11828, ThermoFisher) at 1/200 and anti-GAPDH (Santa Cruz, FL-335) at 1/2000.

### Examination of SK-N-AS CD24 stable cells following Zika virus infection

2.8 x 10^5^ cells of each SK-N-AS clone (WT SK-N-AS, SK-N-AS/VO, SK-N-AS/CD24-v001, and SK-N-AS/CD24-v007) were plated into two wells of a 12-well tissue culture plate. Each sample was then either infected with MOI = 10 of Zika virus strain PRVABC59 or treated with non-infected conditioned media (control). The samples were then incubated at 37°C in 5% CO_2_. After 4 days, the plates were examined under bright field conditions using a Nikon A1R VAAS laser point- and resonant-scanning confocal microscope. After imaging, the cells were collected and counted for determination of Env and NS-1 protein expression by Western blot.

### Viability of CD24 stably-expressing SK-N-AS cells following Zika virus infection

8 x 10^3^ cells were seeded into 12 wells of a 96-well plate for each SK-N-AS sample and controls (WT SK-N-AS, SK-N-AS/VO, SK-N-AS/CD24-v001, and SK-N-AS/CD24-v007). Each sample was then infected with MOI = 10 of Zika virus strain PRVABC59 or treated with non-infected conditioned media (control). Each sample was replicated in sextuplicate. After infection, cells were incubated for 4 days at 37°C in 5% CO_2_. After treatment, a CellTiter 96® Aqueous One Solution Cell Proliferation (Promega Corp.) assay was performed according to the manufacturer’s instructions (PerkinElmer Multilabel Plate Reader–Model 2104) with each well measured in triplicate.

### Caspase 3/7 expression and cell death of CD24-expressing SK-N-AS cells following Zika virus infection

8 x 10^3^ cells were seeded into 12 wells of a 96-well plate for each SK-N-AS sample (including WT SK-N-AS, SK-N-AS/VO, SK-N-AS/CD24-v001, and SK-N-AS/CD24-v007). Each sample was then either infected with an MOI = 10 of Zika virus strain PRVABC59 or treated with non-infected conditioned media (control). Each sample was performed in sextuplicate. After infection, the samples were incubated at 37°C in 5% CO_2_. After 4 days, cell viability was measured by MTS assay. Cell apoptosis was measured by quantifying caspase 3 and 7 production. Caspase-Glo® 3/7 (Promega Corp.) reagent was added to each well, and allowed to incubate at room temperature for 2 hours. Caspase activity was then measured by luminescence using a GloMax luminometer (Promega) with each well measured in triplicate.

### Assess the ability of other Zika virus reference strains to cause lytic infection of CD24-expressing SK-N-AS cells

8 x 10^3^ cells of WT SK-N-AS, SK-N-AS/VO, SK-N-AS/CD24-v001, and SK-N-AS/CD24-v007 were plated in triplicate in a flat bottom 96-well tissue culture plate and allowed to attach overnight. In addition, 2.8 x 10^5^ cells of each were seeded into 12-well tissue culture wells (in quadruplicate) and allowed to attach overnight. The following day, each cell line was infected with MOI = 10 with either Zika virus strains PRVABC59, strain MR766 (ATCC^®^ VR-1838; the Zika virus index strain, derived from a rhesus monkey in 1947, maintained in Vero cells), strain IBH 30656 (ATCC^®^ VR-1839; a 1968 Nigerian human isolate), or treated with non-infected conditioned media (control). All cells were maintained at 37°C in 5% CO_2_. After 4 days, samples in the 12-well plates were examined under bright field conditions using a Nikon A1R VAAS laser point- and resonant-scanning confocal microscope. Samples in the 96-well plate were treated with Viral ToxGlo™ Assay (Promega Corp.) reagents and incubated for 10 minutes. Sample luminescence was then measured using a GloMax luminometer (Promega Corp.). Data presented are a composite of experiments performed in triplicate.

### Statistical analysis

All statistical analyses presented within this manuscript were performed on a minimum of triplicate experimental results. The data were analyzed and combined in Microsoft Excel, then assessed for a p > 0.05 using the Student’s t-test, using one-tailed analysis for changes in viral titer and two-tailed analysis for all other experiments.

## Results

### An array of neuroblastoma cells are permissive to Zika virus infection

**Fig 1:**
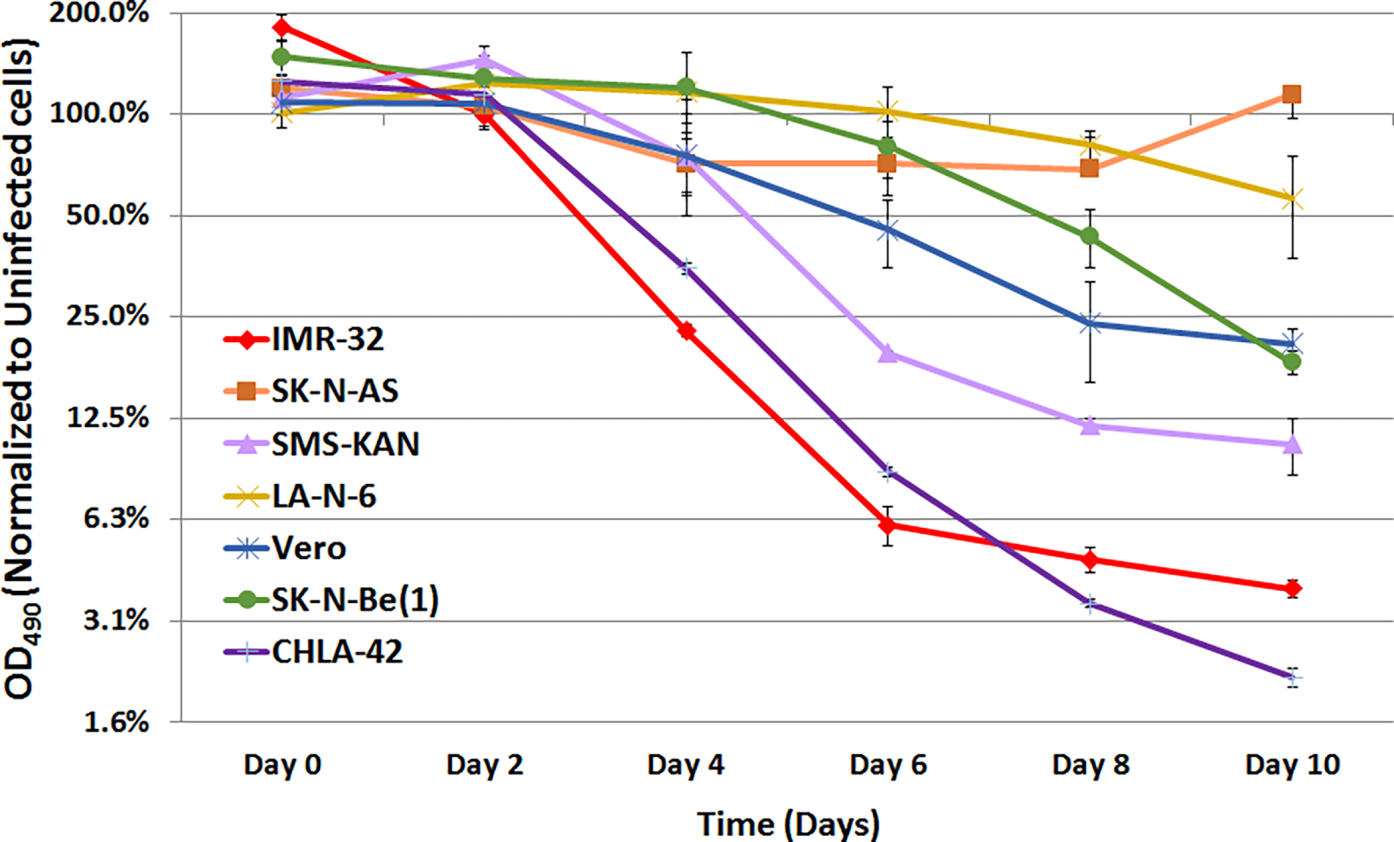
Cell viability time course of neuroblastoma cells infected by Zika virus. Neuroblastoma cell lines IMR-32, SMS-KAN, SK-N-Be(1), SK-N-AS, LA-N-6, and CHLA-42, as well as Vero cells (infection control), were infected with Zika virus strain PRVABC59 and monitored for 10 days. Cell viability was assessed by MTS assay every two days for 10 days total. All infections were performed with MOI = 10 and the results shown are compared to control cells treated with non-infected conditioned media for each cell line. Data shown are the composite of experiments performed in triplicate, with error bars representing standard deviation.

To determine if cultured neuroblastoma cells are permissive to Zika virus infection, we infected a panel of cultured neuroblastoma cells including both MYCN-amplified cells [IMR-32, SMS-KAN, and SK-N-Be(1)] and non-MYCN-amplified cells (SK-N-AS, LA-N-6, and CHLA-42), using Vero cells as infection controls. All cells were infected at an MOI = 10 (strain PRVABC59), and the samples were measured for cell viability every 2 days for a period of 10 days by MTS assay. Reflecting the findings of Hughes *et al*. [30], we found that most, but not all, neuroblastoma cell lines were lysed by Zika virus strain PRVABC59 (Fig 1). In the first few hours after infection, all cell lines tested exhibited increases in cell viability, likely reflecting Zika virus-induced increases in cellular metabolic activity. However, by day 10, nearly all the cell lines showed a decrease in viability of at least 80% (including the Vero control cells). These data suggest that MYCN-amplification was not a distinguishing factor for permissiveness in neuroblastoma. The most surprising result, however, was that Zika virus treatment of SK-N-AS cells showed only a marginal loss of viability initially, but ultimately recovered completely by Day 10. This suggested a possible resistance to Zika viral infection unique to this cell line. Regardless, these observations indicate that a phenotypic range of neuroblastoma cells are susceptible to Zika virus infection, independent of MYCN amplification.

### Zika viruses produce highly permissive infections in all tested neuroblastoma cell lines, except SK-N-AS cells

**Fig 2:**
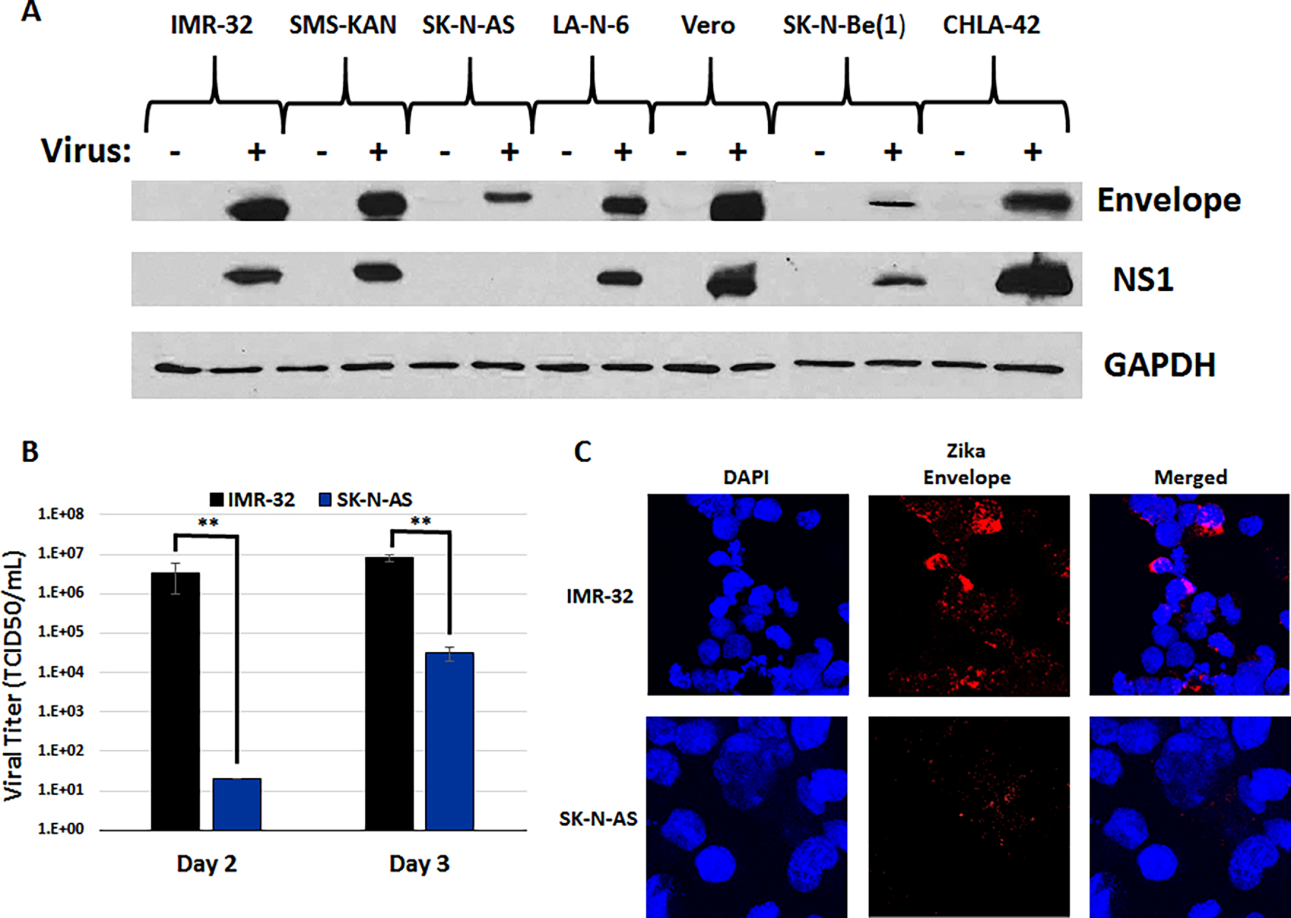
Permissiveness of neuroblastoma cells to Zika viral infection. A) Western blot analysis of Zika virus infections in neuroblastoma cells. Total protein analysis was performed for Zika Envelope protein and NS1 (Non-Structural 1) protein compared to GAPDH control. The neuroblastoma cell lines tested included IMR-32, SMS-KAN, SK-N-AS, LA-N-6, SK-N-Be(1), and CHLA-42, using Vero cells as an infection control. All cells were compared to control cells treated with non-infected conditioned media. Samples were assessed 3 days after infection (MOI = 10). These results were representative of the combined data of experiments performed in triplicate. B) Viral Titer (TCID50) assays of IMR-32 and SK-N-AS cells at Day 2 and 3 post-infection. Data is composed of three biological replicates examined in sextuplicate, with error bars representing standard deviation. ** p > 0.05, Student’s t-test. C) Immunofluorescence labeling of Zika viral Envelope protein in IMR-32 and SK-N-AS cells at Day 3 post-infection. Envelope staining is in red (Alexa Fluor 647) and nuclei are stained in blue (DAPI). Samples are also shown together (merged). Cells were scanned using a Nikon A1R VAAS laser point- and resonant-scanning confocal microscope. Images are at a magnification of 40x with a 4x zoom.

Zika virus binding to exposed cells was examined by demonstrating the presence of cell-associated viral envelope protein. Because the NS1 protein is not expressed until early in the course of infection, Zika virus infection was inferred by demonstration of *de novo* synthesis of the Zika virus NS1 protein. In these experiments, neuroblastoma cell lines were infected with an MOI = 10 of Zika virus particles for four days. At the time of harvest, the culture medium was removed and cells were thoroughly washed in PBS to remove residual virus particles. Total cellular protein was extracted and the viral envelope and NS1 proteins were analyzed by western blot (Fig 2A). In all cases, envelope protein was present in the cell lysates, indicating that Zika virion particles attached to the cell membranes of each cell line. However, a comparison of the NS1 protein levels indicated that all cells expressed detectable levels, with the exception of SK-N-AS cells, that showed no detectable levels of NS1 expression. To determine whether SK-N-AS cells were permissive to Zika viral infection, we compared the culture media viral titers of infected SK-N-AS cells to IMR-32 cells at 2-days and 3-days post-infection (Fig 2B). Viral titer (TCID50) assays confirmed that both cells lines produce active virus; however, IMR-32 viral yields were 5 orders of magnitude greater than SK-N-AS cells (3 x 10^6^ versus 2 x 10^1^) at Day 2 post-infection and remained between 2–3 orders of magnitude greater by Day 3 post-infection (8 x 10^6^ versus 3 x 10^4^). In addition, although immunofluorescent labeling of Zika Envelope protein was robustly detected in IMR-32 cells, the abundance of Envelope protein could barely be confirmed in SK-N-AS cells (Fig 2C). This suggested that the lack of NS1 detection by western blot might have been due to a limit in the sensitivity of the assay due to the poor production of the virus in these cells. Regardless, 3-dimensional Z-stacks confirmed the presence of Envelope protein primarily in the cytoplasm of in IMR-32 cells (S1 Fig). Together, these data indicate that, while Zika virus appears capable of infecting all of the neuroblastoma cell lines tested, SK-N-AS are far less permissive to Zika virus infection, but are still capable of producing low-levels of active virus.

### Permissive Zika virus infection in neuroblastoma cells directly correlates with CD24 expression

Zika virus particles bound to all cell types tested but caused a poorly productive infection in SK-N-AS cells. To determine why Zika virus produced such a poor infection in SK-N-AS cells, we compared the global transcriptomes of the poorly productive SK-N-AS cells to the highly productive IMR-32 cells [33, 34]. We hypothesized that neuroblastoma cell permissivity to Zika virus infection would correlate with expression of a membrane-associated protein. We identified several candidate transcripts encoding cell membrane-associated proteins that are expressed in IMR-32 cells but were poorly expressed in SK-N-AS cells. Among candidates identified were transcripts encoding CD24; CD24 transcripts were highly expressed in IMR-32 cells and minimally expressed in SK-N-AS cells.

CD24 is a glycophosphatidylinositol (GPI)-linked outer membrane sialoglycoprotein anchored to the cell surface and commonly expressed on differentiating neuroblasts, acting as a cell adhesion molecule crucial for neural development [35]. CD24 has been identified as a biomarker for neural lineage differentiation of human stem cells [36] and been shown to directly affect the risk and progression of hepatitis B virus, demonstrating increased risk of more rapid progression to liver cirrhosis and hepatocellular carcinoma [37]. In neuroblastoma, CD24 is often highly expressed and isoforms have been discovered in various tissues during the differentiation process, with their differences often reflecting variations in the extent of their glycosylation [38].

**Fig 3:**
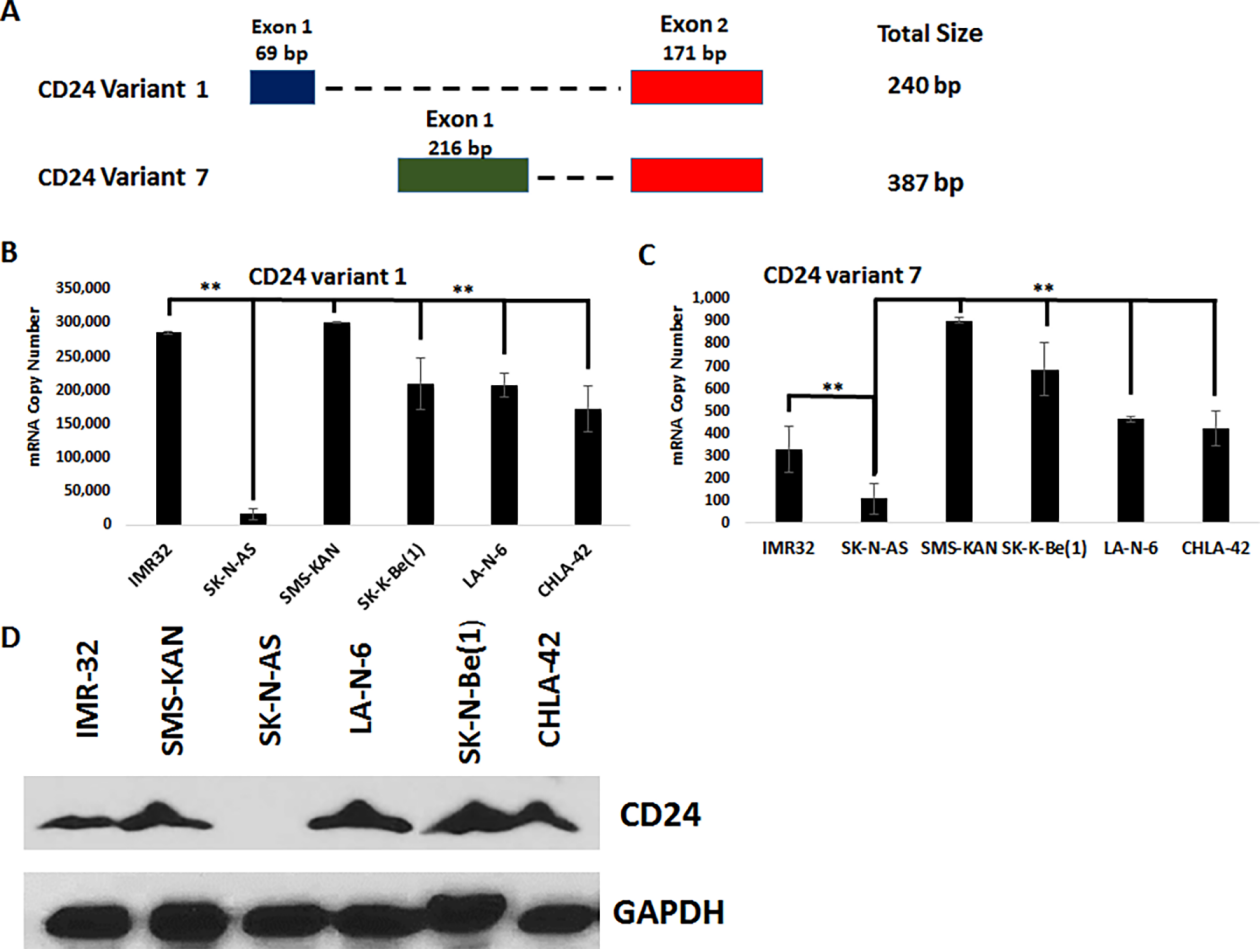
Analysis of CD24 expression in human neuroblastoma cells. A) Schematic of the alignment of CD24 splice variants 1 and 7. B & C) Absolute quantification of CD24 expression by quantitative real-time PCR of total RNA acquired from neuroblastoma cells, measuring CD24 splice variants 1 (B) and 7 (C). Copy number values were normalized to the corresponding GAPDH values to determine the relative copy number. ** p > 0.05, Student’s t-test. D) Western blot analysis of CD24 expression in the total cell lysates of neuroblastoma cells. GAPDH was used as a loading control. All results are representative of the combined data of experiments performed in triplicate, with error bars representing standard deviation.

An analysis of the CD24 transcripts revealed three separate transcripts, all of which were significantly higher expressed in IMR-32 cells than in SK-N-AS cells (S2A Fig). The three transcripts encode two distinct open reading frames, two of the transcripts (NM_013230 & NM_001291738) having the same open reading frame, whereas transcript NM_001291739 utilizes an alternate first exon (S2B and S2C Fig). Both ORFs utilize the same sequence in their second exon. The two splice variants were identified as variant– 001 and variant– 007 (ensembl.org) (Fig 3A). To determine if these CD24 splice variants were expressed in other neuroblastoma cell lines, an absolute quantification of mRNA transcripts was performed using quantitative real-time PCR (qRT-PCR) (Fig 3B & 3C). The results revealed that both CD24 variant– 001 and variant– 007 mRNA transcripts were highly expressed in nearly all neuroblastoma cell lines tested, with the exception of theSK-N-AS cells where the CD24 variants were expressed at very low levels. CD24 expression was validated by western blot analysis of whole cell lysates (Fig 3D). CD24 protein was easily detected in all neuroblastoma cell lines screened, except SK-N-AS cells, in which no CD24 protein was detectable.

### Expression of the tyrosine kinase receptor Axl does not correlate with Zika viral permissiveness of neuroblastoma cells

*Meertens et al* previously reported that *Axl*, a tyrosine kinase receptor, plays a role in promoting Zika viral entry into human microglial cells [39]. RNA-seq data analysis from IMR-32 and SK-N-AS cells showed almost no expression of *Axl* mRNA in the IMR-32 cells and very low expression of *Axl* mRNA in SK-N-AS cells (S3A Fig). This observation was validated by absolute quantification of *Axl* by qRT-PCR (S3B and S3C Fig), comparing *Axl* mRNA expression in the neuroblastoma cell lines to the microglial cell line CHME3 used by *Meertens et al*. The results indicated very robust expression of *Axl* in CHME3 cells (~16000 copies of Axl mRNA per 20 ng of total RNA). However, all neuroblastoma cell lines tested showed very low levels of *Axl* expression, with the most robust being SK-N-AS cells at ~300 copies/20 ng of total RNA and no detectable *Axl* mRNA in IMR-32, CHLA-42, or LA-N-6 cells. Importantly, the pattern of expression did not correlate with susceptibility to Zika virus; poorly permissive SK-N-AS cells expressed significantly more *Axl* mRNA than did all of the highly permissive neuroblastoma cell lines, including IMR-32 cells.

### CD24 expression is necessary for the production of NS1 protein in neuroblastoma cells infected with Zika virus

**Fig 4:**
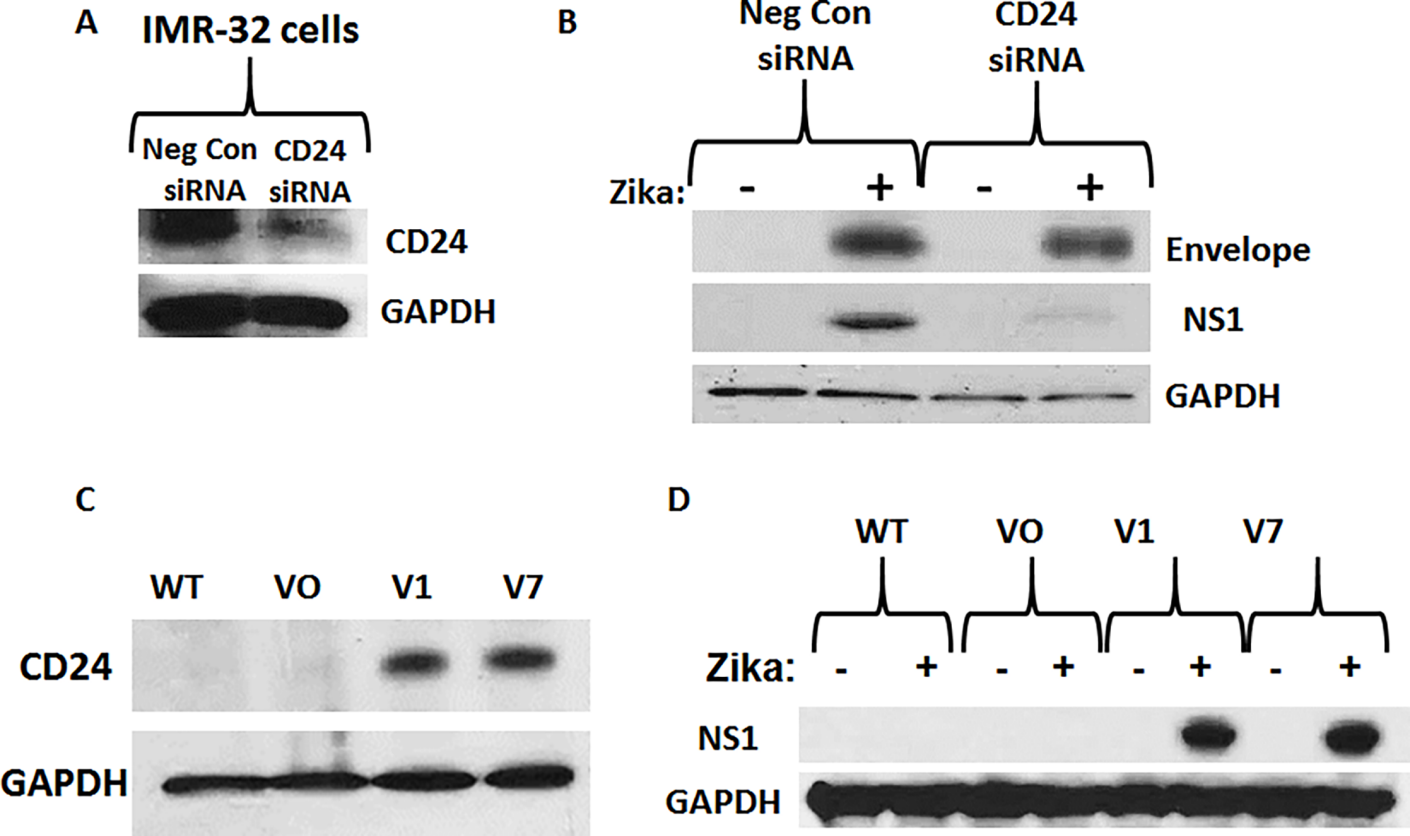
Analysis of the role of CD24 in Zika-virus infected neuroblastoma cells. A) Western blot analysis of siRNA-mediated knock-down of CD24 expression in IMR-32 cells. Samples include Negative Control siRNA and CD24 siRNA. B) Western blot analysis of the expression of Envelope protein and NS1 (Non-Structural 1) protein in IMR-32 cells after siRNA-mediated knock-down of CD24 expression, 96 hours after Zika infection (MOI = 10). Samples include control cells treated with non-infected conditioned media and infected IMR-32 cells transfected with either Negative Control siRNA or CD24 siRNA. C) Western blot analysis of CD24 expression in the human neuroblastoma cell line SK-N-AS, comparing wild type (WT) to stably selected “Vector Only” (VO), CD24 variant 1 (V1), and CD24 variant 7 (V7). D) Western blot analysis of Zika NS1 protein expression 96 hours after Zika infection in CD24-stably expressing SK-N-AS cells, comparing wild type (WT) to stably selected Vector Only (VO), CD24 variant 1 (V1), and CD24 variant 7 (V7). GAPDH was used as a load control for all experiments. All results are representative of the combined data of experiments performed in triplicate.

Given the observation that IMR-32 cells highly express CD24 and are highly permissive to Zika virus infection while SK-N-AS cells do not express CD24 and are poorly permissive to Zika virus infection, we hypothesized that CD24 is a necessary factor for permissive infection of human neuroblastoma cells. To determine if CD24 had any relevance to Zika infection, permissive IMR-32 cells were transfected with a short interfering RNA (siRNA) designed to disrupt expression of all CD24 variant transcripts. The efficacy of the siRNA-induced knock-down of CD24 protein expression in IMR-32 cells is shown in Fig 4A and the effects upon Zika virus infection with strain PRVABC59 (MOI = 10) are shown in Fig 4B. The results indicated that the presence of Envelope protein was largely unchanged after knock-down of CD24; however, the production of NS1 protein diminished considerably, suggesting that CD24 expression was not essential for viral attachment to the host cell, but a minimum threshold of expression was required for the production of the NS1 viral protein. Regardless, siRNA-mediated silencing of CD24 expression reduced expression of viral NS1 protein and prevented Zika virus-induced cell death in IMR-32 cells (data not shown), indicating that CD24 is involved in the increased replication of Zika virus in IMR-32 neuroblastoma cells.

As an alternative means of assessing the need for CD24 in Zika virus infection, both CD24 splice variants 1 and 7 were cloned from cDNA acquired from IMR-32 cells and sub-cloned into eukaryotic expression vectors (pcDNA6/CD24v1 and pcDNA5/CD24v7, respectively). These plasmid constructs were transfected into SK-N-AS cells and expression of the individual splice variants were validated by qRT-PCR using variant-specific primer sets (S4 Fig).

SK-N-AS cells were then selected for stable expression of IMR-32-derived CD24 variants 1 and 7 (Fig 4C). Vector only (VO) cells were also established as a control. Western blot protein analysis results indicated that Zika virus-infected WT and VO SK-N-AS cells failed to express detectable levels of NS1 protein, while both CD24 expressing stable cell lines produce easily detectable levels (Fig 4D). This further suggests that CD24 is necessary for the production of Zika viral NS1 protein in neuroblastoma cells. Bright field images further reveal a striking difference in the pathology between CD24-expressing SK-N-AS cells and controls following Zika infection (S5 Fig). Comparing uninfected and Zika virus-infected cells, we see some loss in both WT and VO cells, correlating with the small initial loss of viability seen previously in Fig 1. Yet, the majority of the cells remain intact. However, both CD24-expressing variant cell lines show dramatic cytopathic effects (CPE) and cell death. These data suggest that stable expression of both CD24 variants 1 and 7 can render SK-N-AS cells highly permissive to Zika virus infection.

### Transgenic CD24 expression in SK-N-AS cells increases Zika virus permissiveness

**Fig 5:**
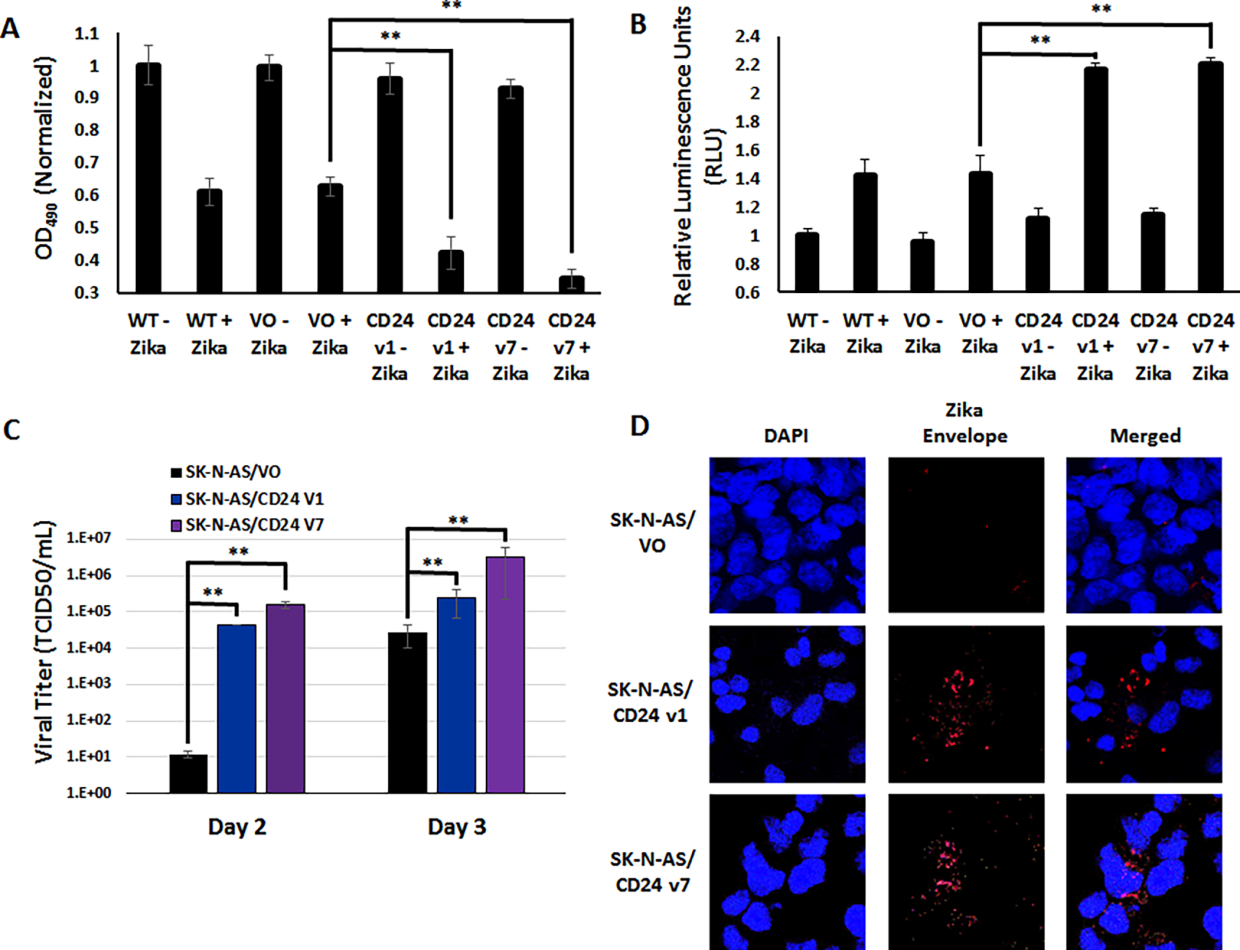
CD24 expression renders SK-N-AS cells susceptible to Zika virus-induced cytotoxicity. A) Cell viability and B) Apoptosis assays comparing Zika virus-infected and uninfected cells. Wild-type (WT) SK-N-AS cells, “Vector Only” (VO) SK-N-AS cells, and SK-N-AS cells expressing CD24 variant 1 (CD24 V1) and CD24 variant 7 (CD24 V7) were infected (MOI = 10) for 96 hours, and then subjected to MTS (Fig 5A) and caspase 3/7 (Fig 5B) assays. Zika infected cells were compared to control cells treated with non-infected conditioned media. The results are representative of the combined data of experiments performed in sextuplicate (n = 6), with error bars representing standard deviation. ** p > 0.05, Student’s t-test. C) Viral Titer (TCID50) assays of SK-N-AS/VO, SK-N-AS/CD24 v1, and SK-N-AS/CD24 v7 cells at Day 2 and 3 post-infection. Data is composed of three biological replicates examined in sextuplicate, with error bars representing standard deviation. ** p > 0.05, Student’s t-test. C) Immunofluorescence labeling of Zika viral Envelope protein in SK-N-AS/VO, SK-N-AS/CD24 v1, and SK-N-AS/CD24 v7 cells at Day 3 post-infection. Envelope staining is in red (Alexa Fluor 647) and nuclei are stained in blue (DAPI). Samples are also shown together (merged). Cells were scanned using a Nikon A1R VAAS laser point- and resonant-scanning confocal microscope. Images are at a magnification of 40x with a 4x zoom.

The robust differences observed in Zika virus permissiveness, as shown in the bright field images in S5 Fig suggest a dramatic change in the phenotype of CD24-expressing cells compared to their control cells after infection with Zika virus. To determine the cause of these differences, the cell samples were examined for changes in cell viability as performed in Fig 1 (Fig 5A). The results indicated that both WT and VO cells infected by Zika virus showed a marked decrease in viability, with losses varying between 30–35% of uninfected cells, consistent with Fig 1. However, the presence of either CD24 variant dramatically decreased viability, ranging from a loss of 60–70% compared to uninfected cells, indicating that cells were undergoing higher states of duress due to CPE. These data were further corroborated with a measurement of the rate of apoptosis (Fig 5B). These results indicated that both WT and VO cells experienced a slight increase in their rates of apoptosis compared to uninfected cells, approximately 40% in both samples. In contrast, the CD24-expressing cell lines showed far more dramatic increases in apoptosis, more than double that observed in control cells (averaging ~ 220% of uninfected cells).

Viral titers within the culture media were also measured from these cells to determine if viral production coincided with cellular pathology (Fig 5C). The results confirmed a startling increase in Zika virus production, with the presence of either CD24 variant 1 or 7 increasing viral titers by ~3–4 orders of magnitude compared to the VO controls at Day 2 post-infection (VO produced only 1 x 10^1^ compared to 4 x 10^4^ for variant 1 and 1 x 10^5^ for variant 7). By Day 3 post-infection, viral titers remained ~10 to 100 fold greater in the CD24-expressing cells compared to controls (2 x 10^4^ versus 2 x 10^5^ or 3 x 10^6^, respectively). Immunofluorescent labeling of Zika Envelope protein remained difficult to detect in SK-N-AS/VO cells, similar to that seen in wild type SK-N-AS cells. However, Zika Envelope protein was prominently expressed in both CD24-expressing stable cell line (Fig 5D) and 3-dimensional Z-stacks again confirmed the presence of Envelope protein primarily in the cytoplasm of these cells (S6 Fig). Together, these data indicate that expression of either CD24 variant 1 or 7 renders SK-N-AS cells highly permissive to Zika virus cytotoxicity and the mechanism of cell death includes induction of apoptosis. In addition, a correlation can be seen between viral pathology and an increase in viral titers produced in the presence of CD24 as well as the production of Zika Envelope protein in CD24-permissive cells.

### Zika virus reference strains MR766 and IBH 30656 induce severe cytopathic effects in CD24-expressing SK-N-AS cells

**Fig 6:**
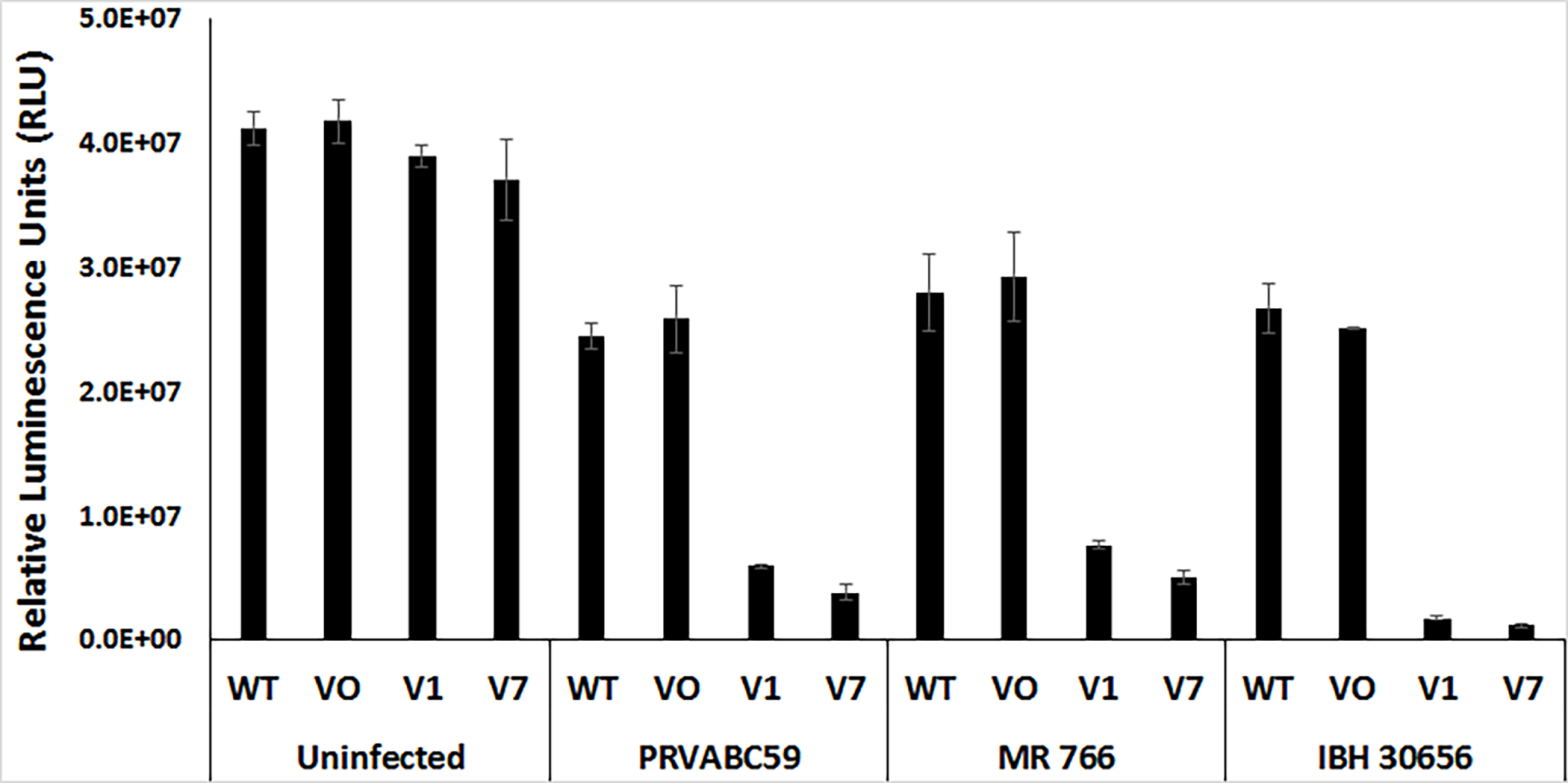
Zika virus strains PRVABC59, MR766 and IBH 30656 all induce severe cytopathic effects in CD24-expressing SK-N-AS cells. Wild-type (WT) SK-N-AS cells, Vector Only (VO) SK-N-AS cells, and SK-N-AS cells expressing CD24 variant 1 (CD24 v1) and CD24 variant 7 (CD24 v7) were infected with Zika virus reference strains PRVABC59, MR 766 and IBH 30656 (MOI = 10). Zika infected cells were compared to control cells treated with non-infected conditioned media. After 96 hours, cellular ATP levels were measured and normalized to cell number. The results are representative of the combined data of experiments performed in triplicate, with error bars representing standard deviation. ** p > 0.05, Student’s t-test.

To confirm that Zika virus-mediated cytotoxicity of CD24 expressing SK-N-AS cells was not limited to the PRVABC59 strain, the cytotoxicity of two additional Zika virus strains, MR 766 (ATCC^®^ VR-1838; the Zika virus index strain, derived from a rhesus monkey in Uganda in 1947) and strain IBH 30656 (ATCC^®^ VR-1839; a 1968 Nigerian human isolate), was also assessed. Zika virus strains PRVABC59, MR766, and IBH 30656 were assessed in parallel for their ability to induce cytopathic effects in CD24 variant 1- and 7-expressing SK-N-AS cells, as well as in wild type and Vector Only SK-N-AS control cells by infecting cells (MOI = 10 for all respective strains) and examining them after 96 hours (Fig 6). Similar to previous experiments, all Zika virus-infected cells exhibited some phenotypic effects after infection. In this case, viral toxicity was determined by comparing the amount of ATP depletion proportional to the number of host cells in culture. CD24-deficient wild-type and Vector-Only cells showed only mild decreases in cellular ATP (equivalent to a loss of ~30–40% compared to uninfected cells), regardless of the strain of Zika virus screened. In contrast, in SK-N-AS cells that stably express CD24, Zika virus strains MR766 and IBH 30656 depleted ATP levels by 75–80% and 90–95%, respectively. Bright field images confirmed significant cytopathic effects and cell death in CD24-expressing cells, regardless of strain (S7 Fig). These results indicate that Zika virus cytopathic effects are far more dramatic in CD24-expressing cells and that this cytopathic effect can be induced even by divergent Zika virus strains.

## Discussion

Neuroblastoma remains a childhood cancer with a disproportionally high mortality rate [2]. Despite survival rates improving for a small subset of patients, the long-term survival for patients with high-risk neuroblastomas remains below 40% [6, 7], illustrating the need for alternative therapeutic approaches. The use of oncolytic viruses for the treatment of neuroblastoma is not a new concept, with various attempts made using measles, adenovirus, poliovirus, and parvovirus [40–43]. Zika virus is the first flaviviruse associated with congenital malformations [23, 24, 44]. The discovery that Zika virus can specifically deplete human neural progenitor cells and impair the growth of human neurospheres correlates with the human congenital Zika virus syndrome [28, 45, 46]. The observations by Hughes et al. and Luplertlop *et al*. that cultured neuroblastoma cells are susceptible to Zika virus-mediated lysis led us to hypothesize that wild-type Zika viruses may be used as an adjunct for the treatment of neuroblastoma in children [29, 30]. Recently, Kaid *et al*. [47] reported the use of a Brazilian Zika virus isolate as an *in vivo* oncolytic treatment for certain human medulloblastoma and atypical teratoid/rhabdoid central nervous system tumors in murine xenograft models. Because most Zika virus infections in children and adults are either asymptomatic or minimally symptomatic, and because Zika virus infections in children and adults have few if any long-term untoward effects [20], we postulated that Zika viruses have evolved to be very specific for the cells that they infect and thus, offer higher specificity with fewer side effects.

Our assessment of the Zika viral treatment of neuroblastoma cells revealed that nearly all cell lines tested were highly permissive to infection. Zika virus infection induced cytopathic effects within days, often leading to apoptosis-induced cell death. A correlation between viral pathology and the production of high viral titers was evident and cytopathic effects could be confirmed in various Zika viral strains, including the Ugandan index strain, MR766. Given that these cytopathic effects were not strain specific, we believe they may represent a common consequence of Zika viral pathogenesis. Likewise, it should be noted that no significant differences were recognized in the pathology between MYCN-amplified versus non-MYCN-amplified neuroblastoma cells, suggesting that MYCN-amplification status may not be a determinant of Zika viral pathogenesis.

Of particular importance was the observation that Zika viruses were cytolytic in every neuroblastoma cell line tested except SK-N-AS. Infection of SK-N-AS cells induced only limited cytopathic effects. Furthermore, and in contrast to infection of the other neuroblastoma lines, Zika virus infection of SK-N-AS cells produced viral titers many orders of magnitude lower than those produced by permissive cells. By identifying cell membrane-associated proteins present in Zika virus susceptible cell lines, but absent in SK-N-AS cells, we implicated CD24 as a factor required for Zika viral permissiveness in neuroblastoma cells. As a GPI-linked glycoprotein commonly found on the surface of differentiating neuroblasts, and a biomarker for neural lineage, we noted that CD24 was also expressed on a wide variety of human tumors [35, 48, 49]. The lack of CD24 expression in SK-N-AS cells renders these cells poorly permissive to Zika infection, evident after complementation with CD24 reversed this phenotype, leading to dramatically increased incidence of CPE and a concurrent increase in viral titer production. This is not the first evidence that CD24 could dramatically improve the progression of a viral pathogen. Previous evidence by Li *et al*. indicated that expression of CD24 increased the risk of both liver cirrhosis and hepatocellular carcinoma caused by hepatitis B virus [37]. Zika virus-susceptible cells express two distinct forms of CD24, both of which complement the defect in Zika viral permissiveness in the previously poorly permissive SK-N-AS cells. Although there is evidence that variations in CD24 isoforms include differences in their isoform glycosylation [38], any differences between these isoforms did not appear to negatively influence the complementation offered to Zika virus.

It is important to note that Meertens *et al*. recently demonstrated that the tyrosine kinase receptor Axl mediates Zika virus entry into human glial cells [39]. Axl is expressed in human microglia and astrocytes of the developing brain. Meertens *et al*. showed that Zika virus entry into microglia and astrocytes requires the Axl ligand Gas6, which acts as a bridge between Zika viruses and the cell surface. Hamel *et al* also showed that both neutralizing antibodies and small interfering RNAs targeting *Axl* expression reduced Zika virus infection in primary dermal fibroblasts [50]. In our analysis of *Axl* expression in neuroblastoma cells we found that an absolute quantification of *Axl* mRNA expression was universally very low and that there was no correlation between *Axl* mRNA expression and susceptibility to Zika virus-mediated lysis or Zika virus infection. Thus, in contrast to microglial and astrocyte cell lines, neuroblastoma cell lines do not appear to require *Axl* expression for Zika virus infection.

If permissiveness to Zika viral infection is dependent on CD24 expression, it is important to note that although a variety of normal human cells express CD24, it is typically expressed at higher levels by metabolically-active cells and by progenitor cells, and at lower levels by terminally differentiated cells [49]. This distinction could help explain why Zika virus infections can be devastating to the developing infant, while being more benign in children and adults. Reflecting on expression of CD24 in normal progenitor cells, CD24 is frequently expressed on human cancer cells [51, 52]. Because CD24 is expressed on the surface of a variety of human tumor cells but is not expressed on most differentiated cells, we propose that therapeutic Zika virus infection of individuals with CD24-positive tumors could result in selective tumor cell infection and lysis, offering a potentially novel use for CD24 as a prognostic marker and Zika virus as treatment. Given the need for alternative therapies in the treatment of high-risk neuroblastoma, the benign side effects of a Zika viral infection use in conjunction with current treatment options can improve outcome and reduce the late effects that can complicate intense tumor treatment. This Zika therapy might also target progenitor cells involved in early relapse, leading to the incorporation of Zika viral therapy into the overall treatment regiment of high-risk neuroblastoma. Similarly, the expression of CD24 on other human tumors offers the prospect for Zika virus treatment of other malignancies, potentially broadening the relevance of these findings to include not only pediatric cancers, but adult tumors as well.

## Supporting information

S1 Fig 3-Dimensional Z-stacks of the immunofluorescent labeling of Zika viral Envelope protein in neuroblastoma cells. Imaging of IMR-32 and SK-N-AS cells was performed at Day 3 post-infection. Envelope staining is in red (Alexa Fluor 647) and nuclei are stained in blue (DAPI). The images presented are merged. Cells were scanned using a Nikon A1R VAAS laser point- and resonant-scanning confocal microscope. Images are at a magnification of 40x with a 4x zoom. Z-stacking was performed using NIS-Elements 4.5 imaging software.
(TIF)

S2 Fig Analysis of Next-Gen (RNA-Seq) sequencing data derived from whole transcriptomes of SK-N-AS and IMR-32 neuroblastoma cells. A) Summary of three discrete CD24 transcripts analyzed from RNA-Seq data. Includes region ID, transcript name (RefSeq), p-values, total reads, RPKM values, and the ratio of fold differences between SK-N-AS cells and IMR-32 cells. B) Analysis of CD24 transcripts by RefSeq and transcript ID, identifying known splice variants. C) Schematic of the alignment of CD24 splice variants in the human genome.
(TIF)

S3 Fig Analysis of Axl mRNA expression in human neuroblastoma cells. A) Summary of *Axl* mRNA transcripts analyzed from RNA-Seq data. Includes region ID, transcript name (RefSeq), *p*-values, total reads, and RPKM values. B) Absolute quantification of *Axl* mRNA expression by qRT-PCR of total RNA (20 ng total RNA/PCR reaction) acquired from neuroblastoma cells. C) Copy number values were normalized to the corresponding GAPDH values to determine the relative copy number. qRT-PCR results are representative of the combined data of experiments performed in triplicate, with error bars representing standard deviation.
(TIF)

S4 Fig Analysis of the ectopic expression CD24 splice variants 1 and 7 after transfection into SK-N-AS neuroblastoma cells. SK-N-AS cells were transfected with the following plasmids, harvested for total RNA after 48 hours, and analyzed by qRT-PCR for the expression of the individual CD24 splice variants: 1) “Vector Only” (VO), 2) CD24 v7,and 3) CD24 v1. A) CD24 variant 1 expression. B) CD24 variant 7 expression. GAPDH was used to normalize the Ct values of each sample, and the relative expression was calculated by normalizing to SK-N-AS/VO cells by ΔΔCt. The results are representative of the combined data of experiments performed in triplicate, with error bars representing standard deviation.
(TIF)

S5 Fig Bright field images of Zika-virus infected CD24-expressing cells and control cells. Control cells were treated with non-infected conditioned media versus Zika infected SK-N-AS cells (MOI = 10, 96 hours after infection) comparing wild type (WT) cells to stably selected Vector Only (VO), CD24 variant 1 (CD24 V1), and CD24 variant 7 (CD24 V7) cells. Images were taken using a Nikon A1R VAAS laser point- and resonant-scanning confocal microscope (40x).
(TIF)

S6 Fig 3-Dimensional Z-stacks of the immunofluorescent labeling of Zika viral Envelope protein in stably selected SK-N-AS cells. Imaging of SK-N-AS/VO, SK-N-AS/CD24 v1, and SK-N-AS/CD24 v7 cells was performed at Day 3 post-infection. Envelope staining is in red (Alexa Fluor 647) and nuclei are stained in blue (DAPI). The images presented are merged. Cells were scanned using a Nikon A1R VAAS laser point- and resonant-scanning confocal microscope. Images are at a magnification of 40x with a 4x zoom. Z-stacking was performed using NIS-Elements 4.5 imaging software.
(TIF)

S7 Fig 3-Dimensional Z-stacks of the immunofluorescent labeling of Zika viral Envelope protein in CD24-expressing SK-N-AS cells. Bright field images of control cells treated with non-infected conditioned media and Zika virus-infected SK-N-AS cells (96 hours after infection) comparing wild type (WT) cells to Vector Only (VO) cells, and to SK-N-AS cells stably expressing CD24 variant 1 (CD24 V1), and CD24 variant 7 (CD24 V7). Infections were performed in tandem for Zika strains PRVABC59, MR766 and IBH 30656 (MOI = 10). Images were taken using a Nikon A1R VAAS laser point- and resonant-scanning confocal microscope (40x). All results are representative of the combined data of experiments performed in triplicate.
(TIF)
