# Supplementary figures and images for: Zika virus as an oncolytic treatment of human neuroblastoma cells requires CD24
Source: PLoS One. 2018 Jul 25;13(7):e0200358. doi: 10.1371/journal.pone.0200358 (PMC6059425; doi:10.1371/journal.pone.0200358)

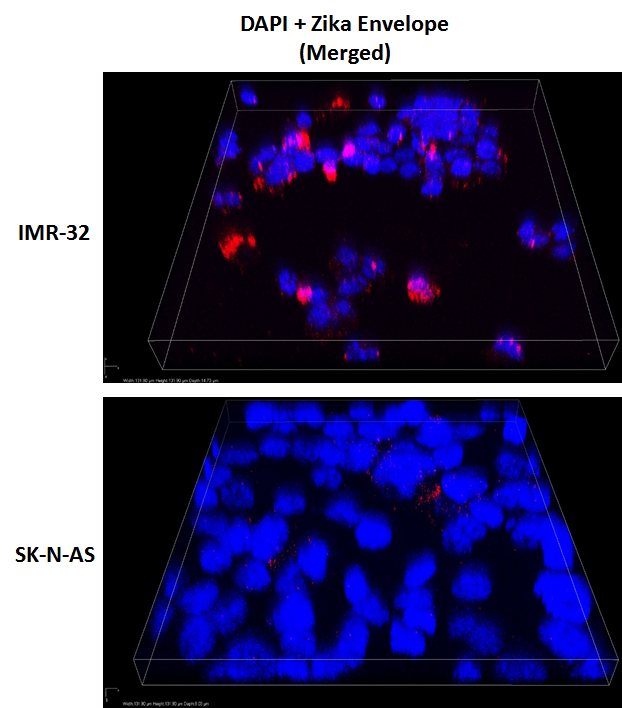

Supplement: S1 Fig — Imaging of IMR-32 and SK-N-AS cells was performed at Day 3 post-infection. Envelope staining is in red (Alexa Fluor 647) and nuclei are stained in blue (DAPI). The images presented are merged. Cells were scanned using a Nikon A1R VAAS laser point- and resonant-scanning confocal microscope. Images are at a magnification of 40x with a 4x zoom. Z-stacking was performed using NIS-Elements 4.5 imaging software. (TIF) [file pone.0200358.s001.tif]

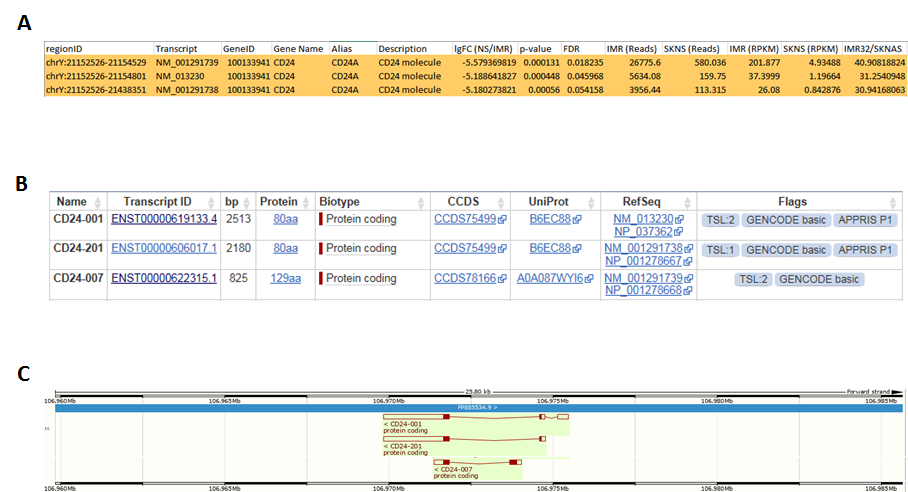

Supplement: S2 Fig — A) Summary of three discrete CD24 transcripts analyzed from RNA-Seq data. Includes region ID, transcript name (RefSeq), p-values, total reads, RPKM values, and the ratio of fold differences between SK-N-AS cells and IMR-32 cells. B) Analysis of CD24 transcripts by RefSeq and transcript ID, identifying known splice variants. C) Schematic of the alignment of CD24 splice variants in the human genome. (TIF) [file pone.0200358.s002.tif]

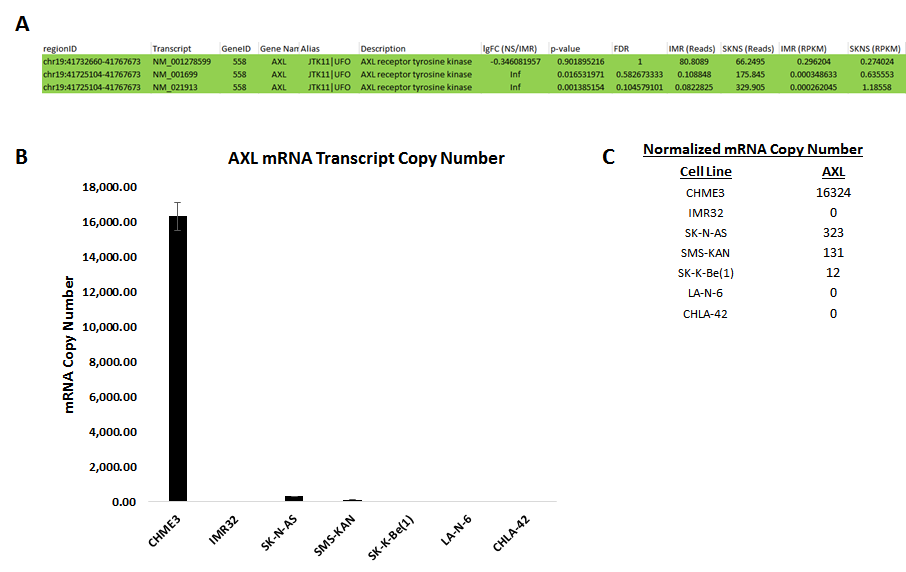

Supplement: S3 Fig — A) Summary of Axl mRNA transcripts analyzed from RNA-Seq data. Includes region ID, transcript name (RefSeq), p-values, total reads, and RPKM values. B) Absolute quantification of Axl mRNA expression by qRT-PCR of total RNA (20 ng total RNA/PCR reaction) acquired from neuroblastoma cells. C) Copy number values were normalized to the corresponding GAPDH values to determine the relative copy number. qRT-PCR results are representative of the combined data of experiments performed in triplicate, with error bars representing standard deviation. (TIF) [file pone.0200358.s003.tif]

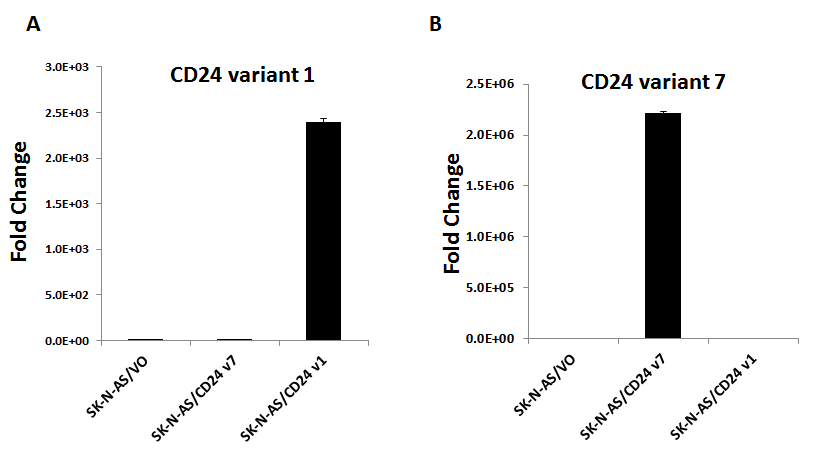

Supplement: S4 Fig — SK-N-AS cells were transfected with the following plasmids, harvested for total RNA after 48 hours, and analyzed by qRT-PCR for the expression of the individual CD24 splice variants: 1) “Vector Only” (VO), 2) CD24 v7,and 3) CD24 v1. A) CD24 variant 1 expression. B) CD24 variant 7 expression. GAPDH was used to normalize the Ct values of each sample, and the relative expression was calculated by normalizing to SK-N-AS/VO cells by ΔΔCt. The results are representative of the combined data of experiments performed in triplicate, with error bars representing standard deviation. (TIF) [file pone.0200358.s004.tif]

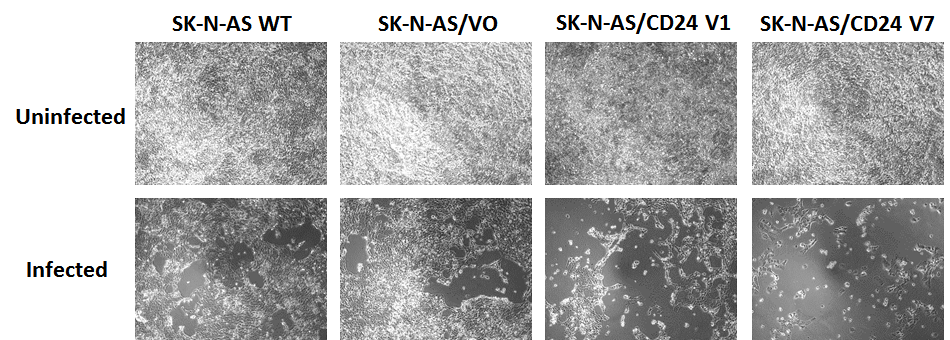

Supplement: S5 Fig — Control cells were treated with non-infected conditioned media versus Zika infected SK-N-AS cells (MOI = 10, 96 hours after infection) comparing wild type (WT) cells to stably selected Vector Only (VO), CD24 variant 1 (CD24 V1), and CD24 variant 7 (CD24 V7) cells. Images were taken using a Nikon A1R VAAS laser point- and resonant-scanning confocal microscope (40x). (TIF) [file pone.0200358.s005.tif]

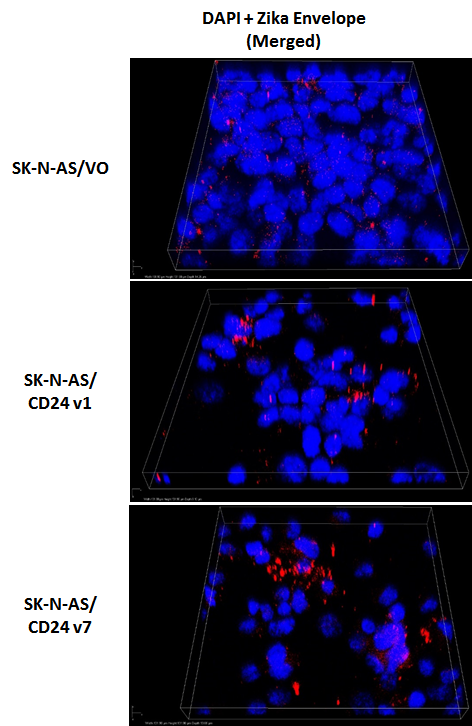

Supplement: S6 Fig — Imaging of SK-N-AS/VO, SK-N-AS/CD24 v1, and SK-N-AS/CD24 v7 cells was performed at Day 3 post-infection. Envelope staining is in red (Alexa Fluor 647) and nuclei are stained in blue (DAPI). The images presented are merged. Cells were scanned using a Nikon A1R VAAS laser point- and resonant-scanning confocal microscope. Images are at a magnification of 40x with a 4x zoom. Z-stacking was performed using NIS-Elements 4.5 imaging software. (TIF) [file pone.0200358.s006.tif]

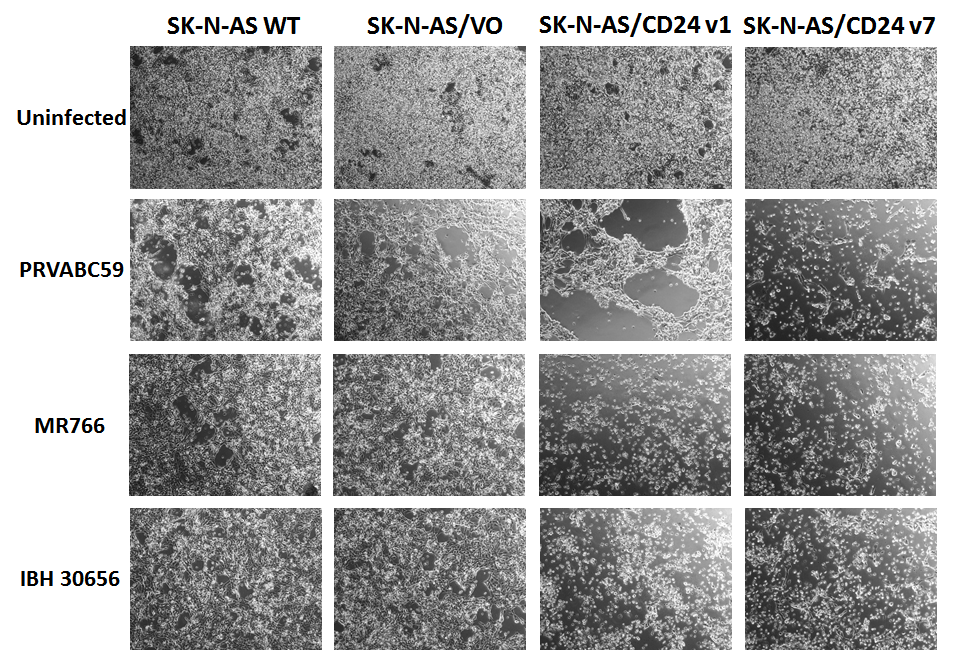

Supplement: S7 Fig — Bright field images of control cells treated with non-infected conditioned media and Zika virus-infected SK-N-AS cells (96 hours after infection) comparing wild type (WT) cells to Vector Only (VO) cells, and to SK-N-AS cells stably expressing CD24 variant 1 (CD24 V1), and CD24 variant 7 (CD24 V7). Infections were performed in tandem for Zika strains PRVABC59, MR766 and IBH 30656 (MOI = 10). Images were taken using a Nikon A1R VAAS laser point- and resonant-scanning confocal microscope (40x). All results are representative of the combined data of experiments performed in triplicate. (TIF) [file pone.0200358.s007.tif]
